# Temporal coupling of cortisol and immune cell responses in acute psychosocial stress

**DOI:** 10.1016/j.bbih.2026.101318

**Published:** 2026-08-27

**Authors:** Hampus Grönvall, Fara Tabrizi, Sylvia Abdelhalim, Jörgen Rosén, Johanna Hirsch, Fredrik Åhs

**Affiliations:** a Mid Sweden University, Östersund, 831 25, Sweden; b Karolinska Institutet, Stockholm, 171 77, Sweden

## Abstract

Acute psychosocial stress triggers coordinated neuroendocrine and immune responses, yet the temporal coupling between cortisol release and leukocyte redistribution in humans remains understudied.

In a sample of 302 participants (aged 18 to 59 years; 64 % female) drawn from the Swedish Twin Registry, we characterized trajectories of neutrophils, lymphocytes, and neutrophil-to-lymphocyte ratio (NLR) following the Trier Social Stress Test (TSST). Blood samples were collected at five timepoints (pre-stress, and at 5, 30, 60, and 90 min after stress), and plasma cortisol was measured at the first three timepoints. Linear mixed-effects models assessed the effects of time, cortisol response (area under the curve with respect to increase; AUC_i_), and covariates (sex, age, body mass index; BMI) on leukocyte dynamics.

The TSST induced canonical biphasic leukocyte responses: lymphocytes peaked 5 min post-stress and declined below baseline during recovery, while neutrophils showed sustained late-phase elevations. Higher cortisol responses were associated with stronger late lymphocyte suppression and higher NLR at 30 to 90 min after stress, but largely unrelated to neutrophil trajectories. Male participants exhibited greater cortisol reactivity and higher late NLR, older age was associated with blunted cortisol and neutrophil recovery, and higher BMI with attenuated early lymphocyte responses.

These findings delineate a plausible phase-specific endocrine–immune coupling in humans where cortisol is linked to variation in lymphocyte redistribution and NLR fluctuations following acute stress. The results suggest a mechanism by which heightened or recurrent HPA-axis activation could influence post-stress inflammatory tone, plausibly contributing to vulnerability for stress-related psychiatric disorders.

## Highlights

• Acute stress triggered biphasic lymphocyte and neutrophil shifts.
• Lymphocytes fell in recovery, neutrophils stayed elevated at 60-90 min.
• Higher cortisol correlated with late lymphocyte drop and higher NLR.
• Sex, age and BMI moderated cortisol and immune trajectories.

## 1 Introduction

Anxiety and depression affect more than 350 million people globally and have been linked with altered immune function and low grade inflammation (Costello et al., 2019; Yuan et al., 2019; Zhang et al., 2026). The immune system consists of several cell types of which neutrophils and lymphocytes are the most common ones. The neutrophil-to-lymphocyte ratio (NLR) is an index that has emerged as a reliable marker of systemic inflammation (Zahorec, 2021). It is practical, accessible and has been associated with cardiovascular, oncological and psychiatric conditions (Brinn and Stone, 2020; Zahorec, 2021; Bhikram and Sandor, 2022; Zeng et al., 2024; Ghafori et al., 2024; Zhou et al., 2025). Chronic stress can change the number of lymphocytes and neutrophils in the circulatory system and is also a major risk for anxiety and depression (Kemeny, 2003; Glaser and Kiecolt-Glaser, 2005). Changes in leukocyte counts have also been observed in post-traumatic stress disorder (PTSD), further supporting the associations between exposure to stress, leukocyte shifts, and psychiatric conditions (Smith et al., 2025). Hence, to characterise how acute stress relates to immune cell dynamics could be of relevance for understanding possible mechanistic links between stress and psychiatric conditions.

The prevalence of anxiety and depressive disorders is greater in women than men, and in younger rather than older adults (Höglund et al., 2020; Solmi et al., 2022; McGrath et al., 2023; Yang et al., 2024; Kayrouz et al., 2025). Another factor that is associated with higher rates of anxiety and depression is adiposity, which can be approximated by the body mass index (BMI) (Jokela and Laakasuo, 2023; Nameni et al., 2024). The associations of sex, age and BMI with anxiety and depression motivates the study of how these demographic factors influence the stress response. A better understanding of how sex, age, and BMI modulate cortisol levels and immune cell counts following stress could reveal whether these factors’ interactions with the stress response help explain their associations with psychiatric disorders.

Acute psychosocial stress activates a cascade of physiological changes. The hypothalamic-pituitary-adrenal (HPA) axis and the sympathetic-adrenal-medullary (SAM) axis are major pathways that control the stress response. These pathways coordinate the release of glucocorticoids and catecholamines, which modulate immune activity (Glaser and Kiecolt-Glaser, 2005; D. H. Hellhammer et al., 2009). The immune response to acute psychosocial stress is biphasic. First, there is a rapid mobilization of immune cells into peripheral circulation, resulting in an increase in neutrophil and lymphocyte counts (Segerstrom and Miller, 2004; Dhabhar et al., 2012). Next, neutrophil and lymphocyte counts quickly return to baseline levels, followed by a sustained increase in neutrophil and decline in lymphocyte counts (Dhabhar et al., 2012; Chan et al., 2023). These two phases in the redistribution of neutrophils and lymphocytes are mediated by neuroendocrine signals, including cortisol and catecholamines (Dhabhar, 2014; Sharma and Farrar, 2020). In murine models, corticotropin-releasing hormone (CRH) has been shown to regulate the early phase independently of adrenocorticotropic hormone (ACTH) and adrenal glucocorticoids, implicating central neural pathways in the initiation of this response (Chan et al., 2023). The later decrease in lymphocytes following stress has been shown to be associated with cortisol release in mice (Poller et al., 2022). In humans, the effect of cortisol on lymphocyte counts is less well studied (Geiger et al., 2015). Although delayed redistribution of neutrophils and lymphocytes has been well-characterized in animal models (Poller et al., 2022), similar patterns in humans remain underexplored.

While many studies have assessed cortisol and immune outcomes independently after acute psychosocial stress, fewer have investigated their dynamic relationship over multiple timepoints (Geiger et al., 2015). Moreover, moderators such as sex, age and BMI may influence both cortisol and immune responses (Wolf et al., 2009; McInnis et al., 2015) but have so far not been investigated in an experimental setting. The present study addresses these knowledge gaps by examining the temporal association between cortisol and immune cell redistribution following acute psychosocial stress. In a large adult sample, we employed the Trier Social Stress Test (TSST), a widely used experimental paradigm for inducing acute psychosocial stress, which reliably elicits HPA axis activation (Kirschbaum et al., 1993; Hellhammer and Schubert, 2012). Blood was sampled at five timepoints across the procedure: pre-stress, immediately following the TSST, and then at 30, 60, and 90 min after stress. Cortisol levels were measured at the first three timepoints, and complete blood counts at all timepoints. We investigated whether cortisol responses predicted changes in neutrophils, lymphocytes and NLR over time, and tested whether sex, age and BMI moderated these associations. By capturing dynamics of cortisol and immune cell responses over time in an experimental setting, we seek to clarify the temporal interactions between stress-induced cortisol release and immune regulation.

## 2 Methods

### 2.1 Participants

As part of a larger project, adult participants were recruited from the Swedish Twin Registry (Zagai et al., 2019). Participants were not selected based on psychiatric or medical diagnoses, and the sample was intended to approximate the general adult population. To allow for a generally population representative sample, the only exclusion criteria were ongoing pregnancy or current severe psychiatric illness. The full sample consisted of 302 twins (78 monozygotic and 53 dizygotic complete pairs, 43 unmatched individuals). Out of these participants, 192 were female and 110 were male. Ages ranged from 18 to 59 years (M = 37.20, SD = 11.00), and BMI ranged from 17.89 to 48.17 (M = 25.34, SD = 4.85). Further sample characteristics (chronic health conditions, on-going medications, socioeconomic status) are reported in Supplementary Material. All participants provided written informed consent prior to participation. The study was approved by The Swedish Ethical Review Authority (Dnr. 2020-00885).

### 2.2 Procedure

All participants were instructed to not consume caffeine or nicotine prior to the session on the day of their participation, as well as to avoid intensive exercise for at least 48 h prior. To account for inter-individual diurnal cortisol variations, all sessions started in the early afternoon. Participants were allowed a brief rest after arriving at the test location at approximately 11:00 – 12:00. Next, height, weight and waist circumference were recorded.

#### 2.2.1 Blood sample collection and management

A peripheral venous catheter was inserted into participants’ non-dominant arm for blood sampling. Venous blood was collected in 5 mL EDTA tubes separately for cortisol and complete blood counts at five timepoints: pre-stress, immediately following the TSST, and then 30, 60, and 90 min post-stress. Pre-stress baseline samples were taken directly after insertion of the catheter. For cortisol analysis, plasma was obtained from one of the samples at the first timepoints by centrifugation at 2000*g* for 10 min and transferred to 5 mL Na-heparin tubes to prevent clotting during transportation. Both the resulting plasma samples and EDTA tubes containing whole blood were stored refrigerated at 4 °C for a maximum of 24 h before transportation to the lab for analyses.

Plasma cortisol concentrations were quantified using ElecSys Cortisol II electrochemiluminescence immunoassays (ECLIA) on the Roche cobas 8000-series platform (Roche Diagnostics, Mannheim, Germany). The assays use a competitive binding principle in which endogenous cortisol competes with a ruthenium-labelled cortisol derivative for binding to a biotinylated monoclonal anti-cortisol antibody. After binding to streptavidin-coated microparticles, electrochemiluminescent emission from the ruthenium complex is measured, with signal intensity inversely proportional to cortisol concentration. Complete blood counts were determined using an Sysmex XN-series automated hematology analyzer (Sysmex Corporation, Kobe, Japan). The instrument combines electrical impedance, photometric analysis, and fluorescence flow cytometry to quantify cell populations. Analyses of cortisol and blood cell count were performed by the Karolinska University Laboratory, Solna.

#### 2.2.2 Stress induction protocol

Participants completed the Trier Social Stress Test (Kirschbaum et al., 1993). They were first given a 3-min preparation period to prepare for a mock job interview to be held in front of two neutral observers. Following the preparation phase, participants were informed that their performance would be video recorded for subsequent evaluation, after which they delivered a 5-min presentation. Immediately afterward, they performed a 5-min mental arithmetic task, counting backwards from 2322 in steps of 13 as fast as they could while being corrected and to start over from the beginning if they made mistakes. Throughout the procedure, the observers maintained neutral expressions and provided minimal feedback. All video recordings were deleted immediately after the session. The entire procedure was performed with participants standing up.

### 2.3 Statistical analyses

Continuous variables were standardised using z-score transformation to allow direct comparisons of effect sizes across predictors. Descriptive statistics were computed for all key variables. Associations between cortisol levels and immune cell outcomes (neutrophils, lymphocytes, NLR) across timepoints were examined using multiple regression analyses, adjusting for age, sex, and BMI.

To investigate cortisol and immune cell trajectories, we first fit linear mixed-effects models for lymphocyte counts, neutrophil counts, the NLR and cortisol separately. Timepoint was used as the main predictor, and age, sex, BMI along with their interactions with timepoint were included as covariates. Random intercepts were included for both participant and twin pairing to account for repeated measures and within-pair clustering. Thus, the base model specification for these analyses of trajectories was:

outcome∼timepoint*(age+sex+bmi)+(1|id)+(1|pairid)

To also examine how cortisol responses were associated with the trajectories of immune cells, we fit extended models for lymphocyte counts, neutrophil counts and the NLR using cortisol area-under-the-curve with respect to increase (AUC_i_) as an additional predictor, as well as interactions with timepoint and age, sex and BMI. Cortisol AUC_i_ was calculated according to Pruessner et al. (2003) using all three cortisol measures, and reflects stress-induced change relative to baseline. The extended model specification was:

outcome∼cortisolAUCi*timepoint*(age+sex+bmi)+(1|id)+(1|pairid)

Because circulating cell counts may be influenced by hemoconcentration, additional models were estimated adjusting for hemoglobin. These models were specified as:

outcome∼cortisolAUCi*timepoint*(age+sex+bmi+hemoglobin)+(1|id)+(1|pairid)

All mixed effects models were estimated using the *lmerTest* (Kuznetsova et al., 2017) package in R, with denominator degrees of freedom approximated via Satterthwaite's method. For each model, explained variance was summarized using marginal R^2^ (variance explained by fixed effects) and conditional R^2^ (variance explained by fixed effects and random effects) using Nakagawa and Schielzeth's method (Nakagawa and Schielzeth, 2013).

To visualise the predicted effects of cortisol response on immune cell trajectories, estimated marginal means (EMMs) were computed with the *emmeans* (Lenth, 2017) package. EMMs were evaluated at representative values of cortisol AUC_i_ – low reactivity (−1 SD), high reactivity (+1 SD), and mean reactivity, averaged across the levels of age, sex and BMI.

To account for multiple comparisons, all reported p-values were corrected using false-discovery rate with the Benhamini-Hochberg procedure, and adjusted p-values < 0.05 were considered significant. Uncorrected p-values for each analysis can be found in their respective supplementary materials.

All analyses were implemented in R version 4.5.0 (R Core Team, 2025) and the *ggplot2* package (Wickham et al., 2024) was used for visualisations.

## 3 Results

### 3.1 Cortisol trajectory following stress

**Fig. 1:**
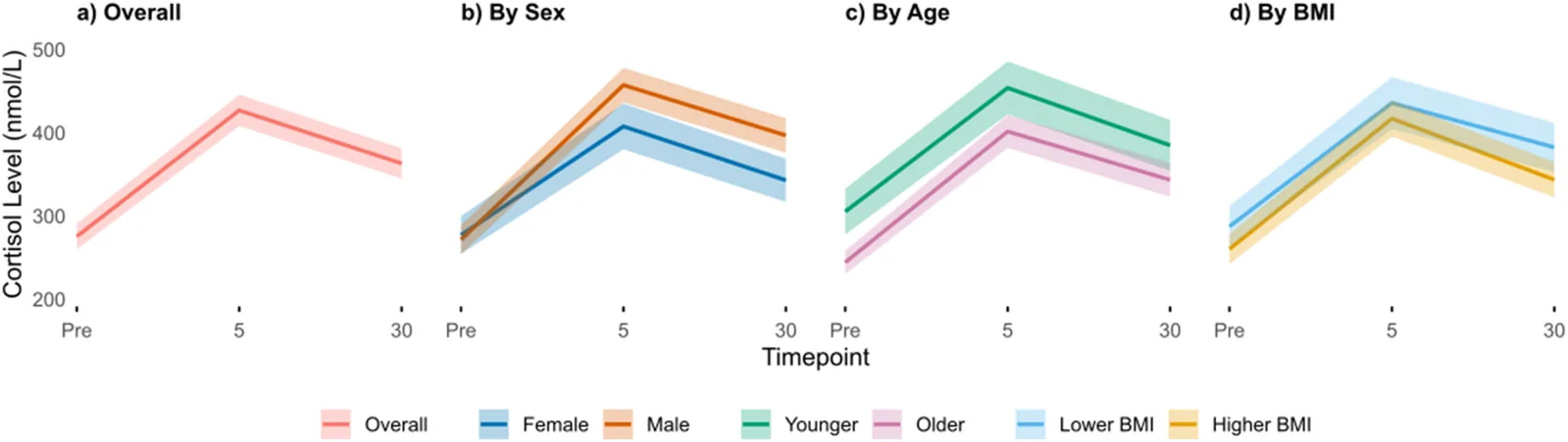
Cortisol levels across the protocol, **a)** overall for the full sample, **b)** separated by participant sex, **c)** separated by age groups (above/below mean age), and **d)** separated by BMI (above/below mean BMI). Shaded region represents 95 % confidence intervals.

As expected, cortisol levels changed significantly across the protocol (*F*_*2,566*_ = 260.86, *p* < .001; Fig. 1a). Fixed-effects estimates confirmed the canonical trajectory, with a sharp increase immediately after the TSST (β = 0.75, *p* < .001) followed by a partial recovery, but remaining above pre-stress levels (β = 0.36, *p* < .001).

While there was no significant main effect of sex, there was a significant interaction of sex and time (*F*_2,566_ = 15.17, *p* < .001; Fig. 1b). Male participants showed stronger cortisol increases from baseline at both 5 min (β = 0.39, *p* < .001) and 30 min (β = 0.41, *p* < .001) after stress. Additionally, a significant main effect of age was observed, with older participants having lower levels of cortisol (*F*_1,174_ = 6.55, *p* = .026; Fig. 1c). There were no significant main or interaction effects of BMI on cortisol levels (Fig. 1d). Observed means and full statistics available in **Supplementary Materials**.

### 3.2 Neutrophil trajectory following stress

**Fig. 2:**
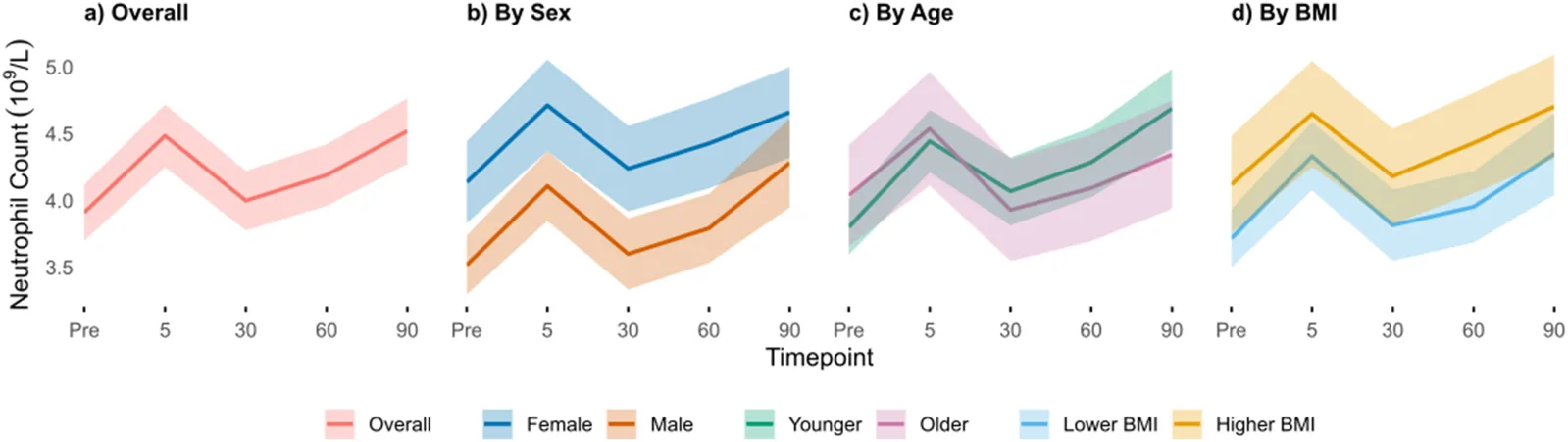
Neutrophil counts across the protocol, **a)** overall for the full sample, **b)** separated by participant sex, **c)** separated by age groups (above/below mean age), and **d)** separated by BMI (above/below mean BMI). Shaded region represents 95 % confidence intervals.

Neutrophil counts strongly changed across time (*F*_4, 1129_ = 79.22, *p* < .001; Fig. 2a). A biphasic pattern was observed, with an initial increase in neutrophil counts at 5 min after stress (β = 0.30, *p* < .001), with a return to near baseline levels at 30 min after stress (β = 0.06, *p* = .08). This was followed by renewed elevation during recovery at 60 (β = 0.16, *p* < .001) and 90 min (β = 0.27, *p* < .001) after stress.

A significant main effect of sex was observed, with female participants showing higher baseline neutrophil counts (*F*_1,178_ = 5.48, *p* = .048; Fig. 2b). There was also a smaller but still significant interaction of sex and time (*F*_4,1129_ = 2.72, *p* = .050), with male participants showing a greater increase from baseline at 90 min after stress (β = 0.11, *p* = .041). Neutrophil trajectories also varied by age, as revealed by a significant interaction of age and time (*F*_4,1130_ = 18.62, *p* < .001; Fig. 2c). Older showed greater declines in neutrophil counts during recovery at 30 min (β = −0.11), 60 min (β = −0.13), and 90 min post-stress (β = −0.17, all *p* < .001). No significant effects of BMI were found (Fig. 2d). Observed means and full statistics available in **Supplementary Materials**.

### 3.3 Lymphocyte trajectory following stress

**Fig. 3:**
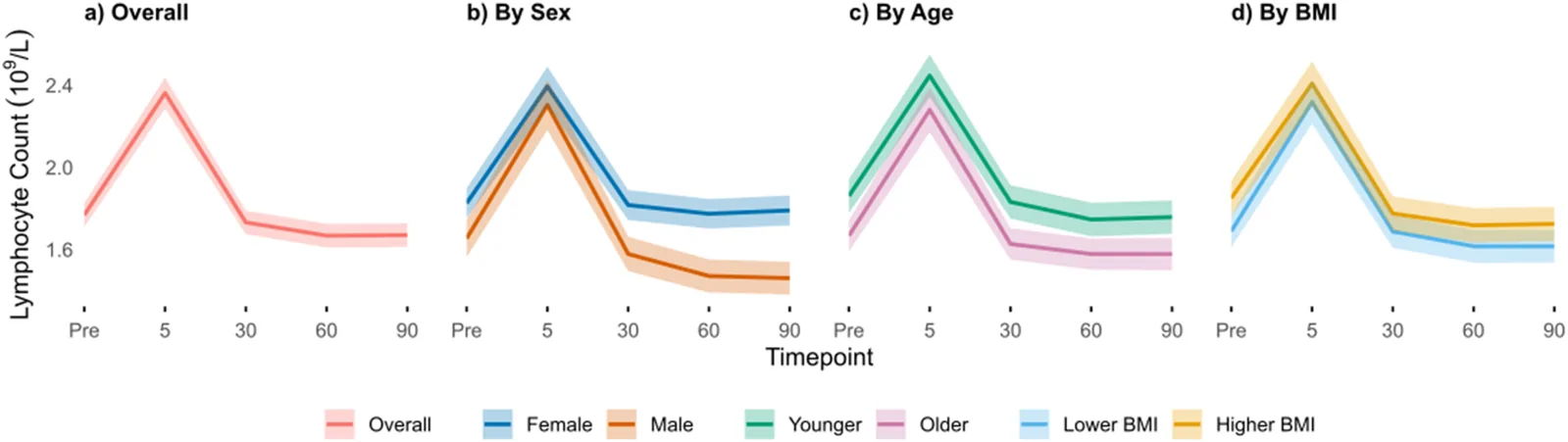
Lymphocyte counts across the protocol, **a)** overall for the full sample, **b)** separated by participant sex, **c)** separated by age groups (above/below mean age), and **d)** separated by BMI (above/below mean BMI). Shaded region represents 95 % confidence intervals.

Lymphocyte counts also changed significantly over time (*F*_4,1130_ = 568.23, *p* < .001; Fig. 3a), with a strong spike at 5 min after stress (β = 0.95, *p* < .001) and a return to baseline at 30 min after stress (β = 0.15, *p* = .729), after which counts stabilized.

A significant main effect of sex was observed, with male participants having lower baseline lymphocyte counts (*F*_1,181_ = 11.76, *p* = .002; Fig. 3b)**.** A significant interaction effect of sex and time also revealed sex-differences in lymphocyte trajectories (*F*_4,1130_ = 14.85, *p* < .001). Specifically, male participants showed a larger early spike at 5 min (β = 0.15, *p* = .043), and greater post-peak declines at 30 (β = −0.12, *p* = .087), 60 (β = −0.21, *p* = .003) and 90 min after stress (β = −0.27, *p* < .001).

Older participants had overall lower lymphocyte counts, independent of time (*F*_1,173_ = 10.61, *p* = .002; Fig. 3c). Additionally, higher BMI was associated with higher overall lymphocyte counts (*F*_1,276_ = 6.77, *p* = .014; Fig. 3d), and a significant time by BMI interaction revealed slight moderation of the trajectory over time (*F*_4,1130_ = 2.95, *p* = .02). Individuals with higher BMI showed smaller increases from baseline in lymphocyte counts at 5 min after stress (β = −0.10, *p* = .003), but no differences at other timepoints were observed. Observed means and full statistics available in **Supplementary Materials**.

### 3.4 Neutrophil-to-Lymphocyte-Ratio trajectory following stress

**Fig. 4:**
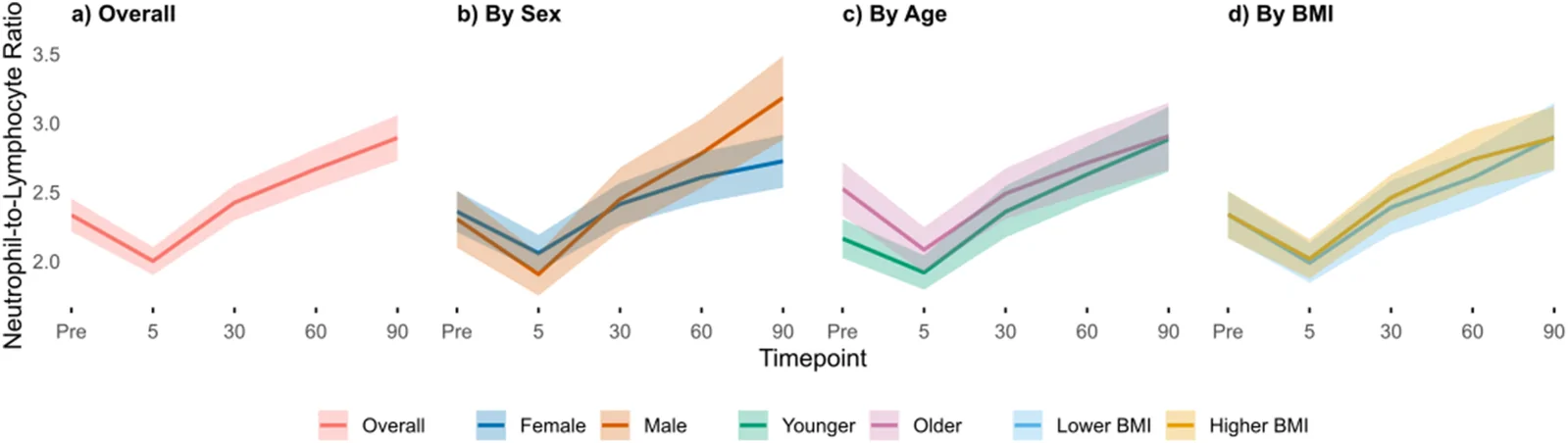
Neutrophil-to-lymphocyte ratio across the protocol, **a)** overall for the full sample, **b)** separated by participant sex, **c)** separated by age groups (above/below mean age), and **d)** separated by BMI (above/below mean BMI). Shaded region represents 95 % confidence intervals.

The neutrophil-to-lymphocyte ratio (NLR) also varied significantly over time (*F*_4,1131_ = 152.94, *p* < .001; Fig. 4a). Compared to baseline levels, NLR was lower 5 min (β = −0.24, *p* < .001), and then returned to baseline at 30 min after stress (β = −0.05, *p* = .334). Following this, the NLR was elevated at 60 (β = −0.22, *p* < .001) and 90 min after stress (β = −0.31, *p* < .001).

A significant interaction effect of sex and time revealed sex differences in NLR trajectories over time (*F*_4, 1131_ = 15.89, *p* < .001; Fig. 4b). While no differences in baseline or the early trajectory were observed, male participants showed greater increases in the late recovery phase at both 60 (β = 0.20, *p* = .012) and 90 min after stress (β = 0.43, *p* < .001).

Furthermore, a significant interaction of age and time showed that NLR trajectories over time differed by age (*F*_4, 1132_ = 6.50, *p* < .001; Fig. 4c). Older individuals showed a greater dip from baseline in the NLR at 5 min after stress (β = −0.10, *p* = .010), which was then followed by blunted increases at 30 (β = −0.13, *p* = .001), 60 (β = −0.14, *p* < .001) and 90 min after stress (β = −0.16, *p* < .001). BMI did not significantly affect the shape or amplitude of the NLR (Fig. 4d). Observed means and full statistics available in **Supplementary Materials**.

### 3.5 Cortisol modulation of blood counts after stress

Multiple linear regression analyses were conducted to examine whether cortisol levels were associated with immune cell counts and NLR at later timepoints while controlling for age, sex, and BMI.

Cortisol level at all timepoints consistently and specifically predicted later counts of lymphocytes and the NLR, while no significant associations were found for cortisol levels and immune cell counts at baseline or 5 min after stress. Higher cortisol at baseline predicted lower lymphocyte counts at 30-90 min post-stress (β = −0.15 to −0.17, *p* ≤ .043), and higher NLR for the same timepoints (β = 0.20 to 0.21, *p* ≤ .006). Cortisol measured at 5 min after stress similarly predicted lower lymphocyte counts (β = −0.18 to −0.29, *p* ≤ .008), and higher NLR (β = 0.18 to 0.35, *p* ≤ .015) at 30-90 min post-stress. Cortisol measured at 30 min after stress predicted lower lymphocyte counts (β = −0.23 to −0.28, *p* < .001) and higher NLR (β = 0.26 to 0.33, *p* < .001) only at 60 and 90 min post-stress. A single cortisol-neutrophil effect was found, with higher cortisol at 5 min after stress being associated with higher neutrophil counts at 90 min post-stress (β = 0.15, *p* = .044). Associations between all remaining time-point combinations were non-significant. Observed means and full statistics available in **Supplementary Materials**.

**Fig. 5:**
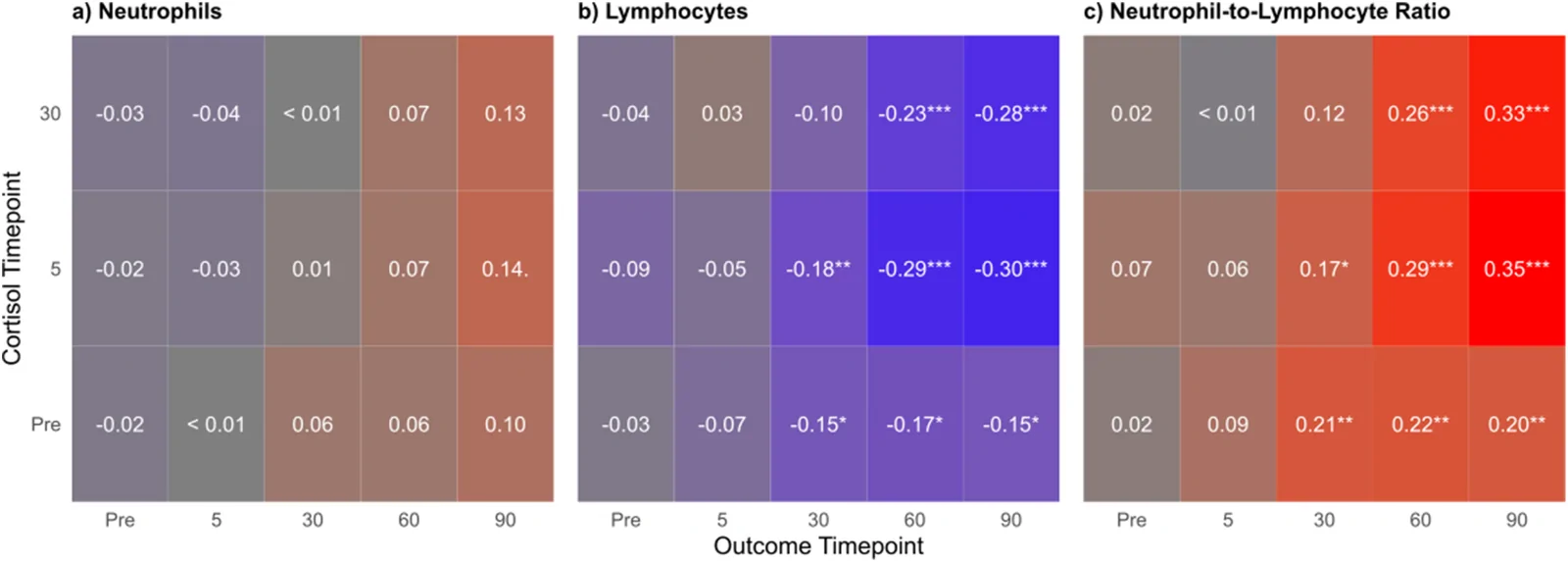
Heatmap of associations between cortisol and **a)** neutrophil counts, **b)** lymphocyte counts and **c)** NLR for each combination of timepoints. Values within cells represent the standardized β-values for cortisol as a predictor. *p < .05, **p < .01, ***p < .001. Red = positive association; Blue = negative association; Gray = null association.(For interpretation of the references to colour in this figure legend, the reader is referred to the Web version of this article.)

Overall, early cortisol responses consistently predicted later lymphocyte counts and NLR, with more limited predictive association for neutrophil counts (Fig. 5).

Next, expanded linear mixed effects models were conducted to evaluate cortisol response modulation of the levels of lymphocytes, neutrophils and NLR. Cortisol area under the curve with respect to increase (AUC_i_) was used to index stress induced cortisol response and included as an additional predictor to the base models previously described.

**Fig. 6:**
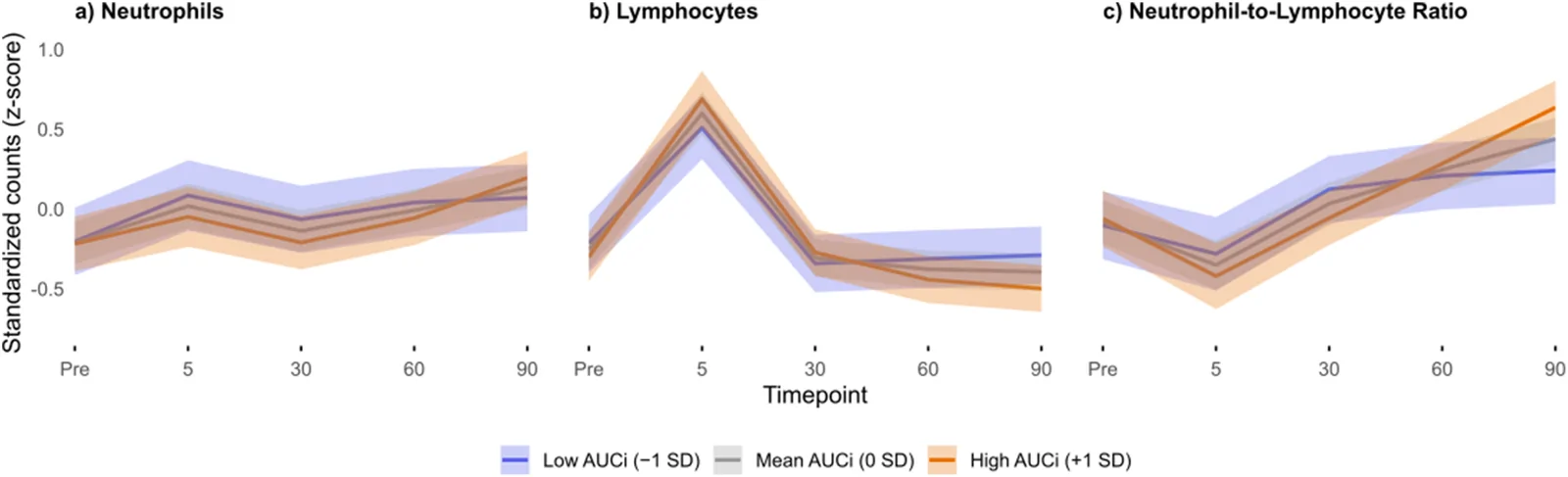
Estimated marginal means (EMMs) of normalized **a)** neutrophil counts, **b)** lymphocyte counts and **c)** NLR. Estimates are visualised for high, low and mean cortisol AUC_i_, averaged across age, sex and BMI. Shaded region represents 95 % confidence intervals.

A significant interaction effect of time by AUC_i_ showed that neutrophil trajectories were modulated by the cortisol response (*F*_4, 1108_ = 7.74, *p* < .001; Fig. 6a). There was a trend toward an association between higher cortisol AUC_i_ and elevated neutrophil counts at 90 min (β = −0.16, *p* = .090). There was no overall association between cortisol AUC_i_ and neutrophil counts (*F*_1, 275_ = 0.21, *p* = .97).

For lymphocytes, there was a significant cortisol AUC_i_ by time interaction (*F*_4, 1109_ = 14.81, *p* < .001; Fig. 6b) indicating that cortisol responses moderated lymphocyte trajectories following stress. Higher cortisol AUC_i_ was associated with a larger early spike at 5 min after stress (β = −0.90, *p* = .051), and greater reduction in lymphocyte counts during late recovery at 60 (β = −0.10, *p* = .037) and 90 min after stress (β = −015, *p* = .001). Thus, greater cortisol responses were associated with more extreme lymphocyte trajectories over time. There was no significant main effect of cortisol response on lymphocyte counts (*F*_1, 282_ = 0.03, *p* = .874).

Finally, the cortisol response related to the shape of the NLR trajectory over time, as revealed by a significant time by cortisol AUC_i_ interaction effect (*F*_4, 1108_ = 18.11, *p* < .001; Fig. 6c). Specifically, higher cortisol AUC_i_ was associated with much higher late-phase NLR at 90 min after stress (β = 0.23, *p* < .001). No main effect of cortisol AUC_i_ on the NLR was observed (*F*_1,282_ = 0.001, *p* = .982), and no other significant effects were found for either outcome measure, see **Supplementary Materials** for full statistics.

### 3.6 Effects of haemoconcentration

To evaluate potential haemoconcentration effects, additional models adjusting for haemoglobin were estimated. Adjustment for haemoglobin did not alter the pattern of results. Both the main effects of timepoint and interaction effects of cortisol AUC_i_ with timepoint remained significant for lymphocytes, neutrophils and NLR (all *p* < .05). For all outcomes, haemoglobin was associated with overall immune cell counts, but no significant interactions with timepoint were observed, indicating that the observed trajectories were not explained by haemoconcentration. Full results are provided in **Supplementary Materials.**

## 4 Discussion

In this study, we investigated the time-courses of neutrophils and lymphocytes in blood after acute psychosocial stress in a large sample. The large sample size allowed evaluation of the influence of individual differences in cortisol, sex, age and BMI on cell counts. We observed the expected canonical biphasic response in immune cell counts, consistent with previous findings (Segerstrom and Miller, 2004; Dhabhar et al., 2012; Chan et al., 2023). Lymphocyte counts initially showed a sharp increase 5 min after stress followed by reduced numbers at 60 and 90 min after stress. Neutrophils also increased acutely following stress but were elevated 60 to 90 min after stress. The stress induced cortisol response was selectively associated with the later phase dynamics of lymphocyte and the neutrophil-to-lymphocyte ratio (NLR). Specifically, individuals with higher stress induced cortisol responses showed larger immediate lymphocyte increases following stress but greater lymphocyte suppression at 60–90 min. While NLR was also increased 60-90 min, this was likely driven primarily by lymphocyte reduction, as late neutrophil trajectories largely independent of the cortisol response, with a significant association only observed for neutrophil counts immediately after stress. Our findings are consistent with a role of HPA-axis activity in the recovery-phase redistribution of lymphocytes.

While cortisol was associated with late lymphocyte dynamics, acute stress responses are jointly mediated by the HPA and sympathetic–adrenal–medullary (SAM) systems. Catecholamines primarily drive rapid leukocyte mobilization via demargination, whereas glucocorticoids are more strongly implicated in delayed redistribution, including lymphocyte egress from circulation (Dhabhar et al., 2012; Sharma and Farrar, 2020). The observed biphasic pattern likely reflects these temporally distinct but interacting processes, rather than cortisol alone. Studies in humans have found negative associations between baseline cortisol and lymphocyte counts as well positive correlations between cortisol and neutrophils (Cole, 2008), but less is known about the time course of acute stress’ influence on these relationships. Recent studies in mice show that central motor and fear circuits acutely mobilize neutrophils independently of cortisol, while CRH-mediated HPA signalling drives delayed lymphocyte redistribution (Poller et al., 2022; Chan et al., 2023). The lack of a strong cortisol effect on neutrophil counts in our sample is consistent with other factors, rather than cortisol, governing neutrophil demargination following stress (Poller et al., 2022). Our observations that cortisol response early after stress predicted later lymphocyte counts and NLR, together with our finding that cortisol response selectively moderated lymphocyte and the resulting NLR during recovery, are consistent with patterns of glucocorticoid-related redistribution of lymphocytes from the blood stream and extends on previous research that only examined single or sparse timepoints (Kirschbaum et al., 1993; Wolf et al., 2009; Hellhammer and Schubert, 2012; Etzel et al., 2022).

We also observed that the cortisol-immune relationships were moderated by sex, age and BMI. Men showed a greater cortisol response after stress than women which aligns with a previous study in 282 participants (Stephens et al., 2016). Our study also fills a previous gap in the literature regarding sex differences in leukocyte counts following acute stress (Bekhbat and Neigh, 2018). We show that lymphocyte counts were lower in males at 60 to 90 min post stress, while NLR was higher, which may reflect sex specific hormonal or receptor-level differences that influence how cortisol or catecholamines act on immune cells (Slavich and Sacher, 2019). This is concordant with our results showing that increased cortisol response was negatively correlated with lymphocyte counts overall.

Increasing age was also associated with lower cortisol levels, which mirrors a previous stress study comparing older and younger groups of adults (Mikneviciute et al., 2023). Ageing has also been associated with lower baseline cortisol levels especially in individuals over 40 years (Ahn et al., 2007). In our sample, older age was associated with reduced neutrophil counts in the late phase after stress and lower recovery of NLR. In contrast, lymphocytes response was not affected by age, though overall counts of lymphocytes were lower in older adults. Hence, age modulated stress induced neutrophils more than lymphocytes, causing a shift in the balance between these cell types. These results align with age related reductions in immune responsivity and flexibility (Segerstrom and Miller, 2004).

The BMI findings suggest a dampening of cortisol's immunomodulatory effect in individuals with higher adiposity. Participants with elevated BMI had a smaller lymphocyte surge immediately after stress, and overall, their cortisol responses were less correlated with immune cell changes. This pattern is consistent with the concept of glucocorticoid resistance in obesity (McInnis et al., 2015) and highlights adiposity as a modifier of stress-immune regulation.

Because leukocyte counts are concentration-based measures, blood volume shifts could theoretically influence observed cell numbers. However, haemoglobin adjustment did not alter the temporal trajectories of immune cells or the cortisol-related interactions, and haemoglobin did not vary systematically over time. Taken together, this suggests that the observed leukocyte dynamics reflect a redistribution of cells that is not primarily explained by haemoconcentration.

### 4.1 Implications for psychiatric disorders

Acute stress responses are adaptive, but individual differences in their magnitude and recovery may be relevant for psychopathology. Dysregulated response such as exaggerated reactivity, delayed recovery, or reduced glucocorticoid sensitivity may bias immune signalling toward a more pro-inflammatory profile over time (Dhabhar, 2014; Miller and Raison, 2016). The NLR integrates neutrophil and lymphocyte counts into a composite measure of peripheral inflammation, which has been linked to depression and other psychiatric conditions as a possible predictive biomarker (Bhikram and Sandor, 2022; Cheng et al., 2022; Ghafori et al., 2024). In individuals with Post-Traumatic Stress Disorder, the proportions of B-cells and NK-cells have been found to be lower while the proportion of neutrophils have been found to be higher than in people without the disorder (Smith et al., 2025). Further, genetic liability for depression is associated with increased counts of leukocytes, suggesting that heightened leukocyte counts, but still counts within normal range, can be a vulnerability factor for depression (Sealock et al., 2021).

Acute and chronic stress are distinct, and transient changes such as those observed here are not equivalent to persistent inflammation. However, chronic stress likely reflects repeated or insufficiently resolved acute responses. If recovery processes are impaired or repeatedly engaged, these short-term immune fluctuations may accumulate and contribute to longer-term alterations in immune regulation (Segerstrom and Miller, 2004; Dhabhar, 2014). Our findings that a single exposure to psychosocial stress produced a cortisol-coupled NLR elevation in the late phase suggests a plausible mechanism for stress-triggered inflammatory bias (Dantzer et al., 2008; Miller and Raison, 2016; Bower and Kuhlman, 2023). Repeated activation, especially in high cortisol responders or individuals with glucocorticoid resistance, could cumulatively heighten inflammatory tone and contribute vulnerability to psychopathologies (Ravi et al., 2021).

### 4.2 Limitations

Cortisol was sampled only at three out of five timepoints. The lack of measurements at 60 and 90 min post-stress limits precision in modelling late endocrine-immune coupling. Further, although the TSST provides experimental control over the stressor, we did not include a control condition where samples were collected at the same timepoints without stress. However, the temporal ordering (cortisol at early timepoints correlate with later immune cell counts, but not vice-versa) strengthens plausibility.

### 4.3 Conclusions

Acute psychosocial stress elicited the canonical biphasic leukocyte response, with diverging time-courses for lymphocytes and neutrophils 60 and 90 min after stress. Inter-individual differences in cortisol output were primarily associated with the recovery phase, manifesting as lower lymphocyte counts and a higher NLR at 60 and 90 min after stress among high responders. Lymphocyte counts were also influenced by sex, age and BMI suggesting neutrophil dynamics were robust and largely cortisol-independent, whereas sex, age, and BMI modestly moderated neutrophil trajectories. These findings delineate phase-specific endocrine–immune coupling in humans and suggest a pathway by which repeated stress exposure could bias inflammatory tone. Although the acute stress response is adaptive, individual differences immune cell dynamics can be relevant for understanding risk for psychopathology. Future studies in large samples of people with and without psychiatric disorders could incorporate broad panels of proteins, metabolites and gene expression to determine pathways that could serve as new treatment targets. In sum, our time-resolved analyses clarify how HPA-axis reactivity calibrates late immune redistribution after stress, refining mechanistic accounts of stress–inflammation links relevant to psychiatric risk.

## CRediT authorship contribution statement

**Hampus Grönvall:** Writing – review & editing, Writing – original draft, Visualization, Software, Methodology, Investigation, Formal analysis, Data curation, Conceptualization. **Fara Tabrizi:** Writing – review & editing. **Sylvia Abdelhalim:** Writing – review & editing. **Jörgen Rosén:** Methodology, Conceptualization. **Johanna Hirsch:** Writing – review & editing. **Fredrik Åhs:** Writing – review & editing, Supervision, Project administration, Funding acquisition, Conceptualization.

## Ethics declaration

Written informed consent to take part in the study and to publish the article has been obtained from all participants or their legal representatives. The privacy rights of participants have been observed.

This study included organ or tissue donors. This study includes human biological material and consent was obtained by donors, or their next of kin or legal representatives, for use in this study and for publication of the article. The samples used in this research were not sourced from executed prisoners or prisoners of conscience.

This study was performed in compliance with relevant laws, regulatory frameworks and guidelines where the research took place. This study was approved by the Etikprövningsmyndigheten. (Approval No. Dnr. 2021-00885)

## Funding

This work was supported by the Swedish Research Council (grants No. 2018-01322 and 2023-01093) and the bank of Sweden Tercentary Foundation (grant No. P25-0219).

## Declaration of competing interest

The authors declare no conflict of interest.

## Data Availability

Data will be made available on request.
